# Fiber optic micro sensor for the measurement of tendon forces

**DOI:** 10.1186/1475-925X-11-77

**Published:** 2012-10-03

**Authors:** Gregory P Behrmann, Joseph Hidler, Mark S Mirotznik

**Affiliations:** 1 Department of Electrical Engineering, Catholic University of America, Washington, DC, 20064, USA; 2 Aretech LLC, Ashburn, VA, 20147, USA; 3 The University of Delaware, Newark, DE, 19716, USA

**Keywords:** Fiber Bragg grating sensor, Tendon forces

## Abstract

A fiber optic sensor developed for the measurement of tendon forces was designed, numerically modeled, fabricated, and experimentally evaluated. The sensor incorporated fiber Bragg gratings and micro-fabricated stainless steel housings. A fiber Bragg grating is an optical device that is spectrally sensitive to axial strain. Stainless steel housings were designed to convert radial forces applied to the housing into axial forces that could be sensed by the fiber Bragg grating. The metal housings were fabricated by several methods including laser micromachining, swaging, and hydroforming. Designs are presented that allow for simultaneous temperature and force measurements as well as for simultaneous resolution of multi-axis forces.

The sensor was experimentally evaluated by hydrostatic loading and *in vitro* testing. A commercial hydraulic burst tester was used to provide uniform pressures on the sensor in order to establish the linearity, repeatability, and accuracy characteristics of the sensor. The *in vitro* experiments were performed in excised tendon and in a dynamic gait simulator to simulate biological conditions. In both experimental conditions, the sensor was found to be a sensitive and reliable method for acquiring minimally invasive measurements of soft tissue forces. Our results suggest that this sensor will prove useful in a variety of biomechanical measurements.

## Introduction

Our understanding of the neuromuscular control system in healthy people and in those with disabilities would be significantly enhanced by using in vivo tendon force measurement methods. By measuring bone-soft tissue interface forces, we could enhance the control algorithms for prosthetic devices. Additionally, such data would significantly extend our knowledge of tissue mechanics, biomechanics and orthopedics.

Currently, most soft tissue force measurements are estimated indirectly using biomechanical models that approximate the actual system. If the forces could be measured accurately and in natural settings, and then combined with imaging devices such as MRI, we could completely characterize tissue properties such as stress–strain relationships [1].

The aim of this study was to investigate the design, fabrication and characterization of a novel fiber optic sensor whose primary application is the measurement of tendon forces. The sensor incorporates fiber Bragg gratings and micro-fabricated stainless steel housings. A fiber Bragg grating is an optical device that is spectrally sensitive to axial strain. Stainless steel housings are designed to convert radial forces that are applied to the housing into axial forces that can be sensed by the fiber Bragg grating. This sensor offers several advantageous properties. First, it is smaller and less invasive than traditional tendon force sensors. Second, by measuring spectral response, it is insensitive to transmission losses caused the optical fiber bending away from the measurement area of interest. Third, the housing design allows us to incorporate a temperature sensing reference. Finally, the use of multiple wavelengths or housings with a preferential orientation, allows us to resolve or isolate multi-directional forces.A major portion of this effort involves the design, modeling, and fabrication of stainless steel housings that are capable of converting radial forces into axial forces that can be exerted on a fiber Bragg grating. Three fabrication methods are investigated for their suitability in providing the desired housing shape. These methods are laser micromachining, swaging, and hydroforming. An assembly method is developed as well as a calibration method. Finally *in vitro* testing is performed on excised tendon.

## Background

### In vivo *force measurements*

#### *Indirect Estimation Techniques of* In Vivo *Tendon Forces*

Currently, the primary means of estimating forces transmitted through tendons are indirect, mathematical techniques [1]. In most experimental preparations, single or multi-degrees of freedom load cells are used to measure joint torques, where the measured torque results from all the muscles and soft tissues that span the joint. Estimates of moment arms from cadavers coupled with measurements of the muscle’s physiological cross-sectional area are used to perform static and dynamic optimization procedures in order to distribute the forces across each of the muscles at the joint of interest. Combining this information with imaging techniques that approximate the muscle insertion points on the different tendons gives estimates of the tendon forces.

These estimation techniques are severely limited by a number of factors. The torque generated by each individual muscle is highly dependent on the magnitude of its moment arm [2], so attempts to scale generalized moment arm relationships derived from cadaver studies to different sized subjects creates a great deal of numerical error. Furthermore, under dynamic conditions such as during gait, muscle behavior is highly non-linear and time-varying, characteristics not reliably captured by current muscle models. Finally, the mathematical complexity involved in attempting to model every muscle spanning a joint and the indeterminacy of redundant actuators require simplifications to be made regarding the number of muscles represented in the optimization procedure. This lumping of muscles into clusters usually leads to muscle forces being overestimated, and consequently the magnitude and concentration of forces acting on and through the tendon being biased.

### Direct estimation of tendon forces

In 1969, Salmons introduced a technique for quantifying tendon forces directly using a buckle transducer. This buckle transducer, also used later by others [3-7] to measure tendon forces, was nothing more than a small mechanical buckle instrumented with strain gauges that responded to tension in the tendon. Due to the size of the buckle transducer, this technique has only been used to study the Achilles tendon due to this tendon’s accessibility and size. Results from these studies have offered some of the most compelling insights into tendon behavior under natural conditions, including information on the loads that are generated during walking, running, and other dynamic conditions.

The primary limitation of this technique is the invasiveness of introducing and removing the sensor. The transducer is implanted under local anesthesia with an incision approximately 50 mm long made on the lateral side of the Achilles tendon. Wires from the strain gauges are brought outside the skin and covered with a sterile dressing. Normal activity cannot be resumed for 2–3 weeks so the wound can heal completely. Clearly such an invasive procedure would be impossible to introduce into single session experiments. Furthermore, complications with these buckle transducers, such as infections and pain, have limited the use of these sensors to a small and restricted research population.

A number of studies have looked into utilizing fiber optics as a means of sensing in vivo tendon forces [8-13]. The primary advantage of this technique is that the fibers are very small (approximately 200μm), reducing the invasiveness caused by introducing the sensor into the tendon. Furthermore, optical sensors can be readily incorporated into imaging devices such as MRI and CT, allowing experimenters to monitor both kinetic and kinematic variables simultaneously [9]. In [9] Komi et. al. demonstrated that in reduced rabbit preparations, the fiber optic sensor output was very/highly linear with increasing tendon loads (r = 0.999). They further demonstrated that the fiber was able to track dynamic loading conditions and that the fiber measurements were repeatable. Erdemir et. al. [11-13] further tested the application of optical sensors for measuring tendon forces, and addressed the effect of skin on the transducer output and force predictions. Using human cadaver feet as the test preparation and the same fiber configuration as in [9], they found that skin movement, loading rate, and cable migration all influenced the accuracy of the optical sensor. These early experiments indicate that fiber optical sensing of tendon forces may be a viable option for in vivo human testing if artifacts such as “skin effects”, sensor placement, and proper calibration can be addressed.

### Background on optical force transducers

The basic idea behind a fiber optical force sensor is that an external pressure exerted on the surface of the fiber will cause a deformation that can be measured by the resulting change or modulation of the light signal that passes through the fiber. There are two methods that have chiefly been used to produce this effect: intensity based and spectral based optical force sensors.

### Intensity based optical force sensing

**Figure 1:**
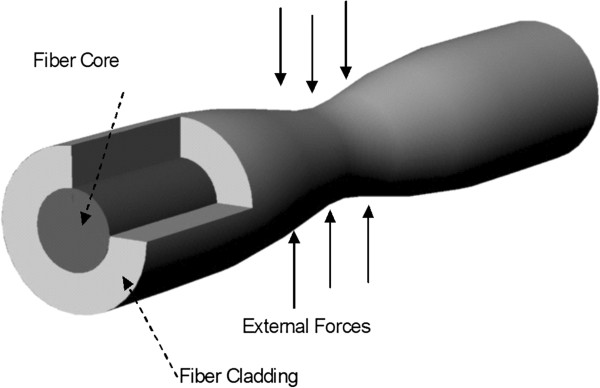
Illustration of the use of bending or pitching of an optical fiber to be used as a force sensor.

One of the simplest yet effective methods for constructing a fiber optic force sensor involves modulating the intensity of the optical beam as a function of the external forces. In this method, when the surface of the fiber is subjected to forces, the result is an elastic deformation, such as compression, bending or twisting. This causes the following: (1) some of the light exits the core into the cladding and leaks outside the fiber, and/or (2) some of the light gets reflected from the location of deformation back towards the source (see Figure 1). Both phenomena lead to the same overall effect, namely, an attenuation of the light intensity arriving at the opposite end. Conceptually, this resembles pinching the middle of a water hose to reduce the flow. The degree of this light attenuation can be used to indirectly measure the amount/magnitude of the external deformation forces. Komi et. al. [9] used this technique with multimode bare plastic optical fibers (POF) of 0.3-0.5 mm diameters to investigate tendon forces in rabbits and humans up to 6000 N. Here the fiber was inserted through the skin, through the entire tendon, then through the skin and outside the body. One end of the fiber was attached to a light source and the other was attached to a detector. Forces were applied to the tendon and transmission losses were measured. Measurements of the incident and transmitted light intensities allowed quantification of the external forces.

The main advantages of this approach lie in the ease of inserting the sensor and the low cost. However, heavy, static loading has led to permanent bending of the plastic fiber [12,13]. More importantly, in full in vivo measurements, optical losses due to tendon forces and losses due to bending of skin surrounding the tendon have been difficult to differentiate [12-14].

### Spectral based optical force sensing

All optical fibers have wavelength dependent or spectral properties called dispersion. Thus, another approach for constructing a fiber optic force sensor is altering the fiber’s spectral properties as a result of mechanical deformation. This approach offers the advantage of being resistant to errors that are induced by intensity variations such as cable bends and connector losses. In this study we investigated a special spectral based sensor called a fiber Bragg grating (FBG) [15]. FBGs are very mature products that have been used in civil engineering applications for over a decade to accurately measure forces in bridges, robotic actuators [16,17] and other structures.

A conventional fiber Bragg grating consists of a periodic modulation of the index of refraction in the core of a single mode fiber optic cable. If a fiber Bragg grating is illuminated with broadband light that meets the Bragg condition, reflected light from each grating plane adds constructively and results in a narrow back-reflected center wavelength. This condition is expressed by Equation 1.

(1) λB=2neffΛ

**Figure 2:**
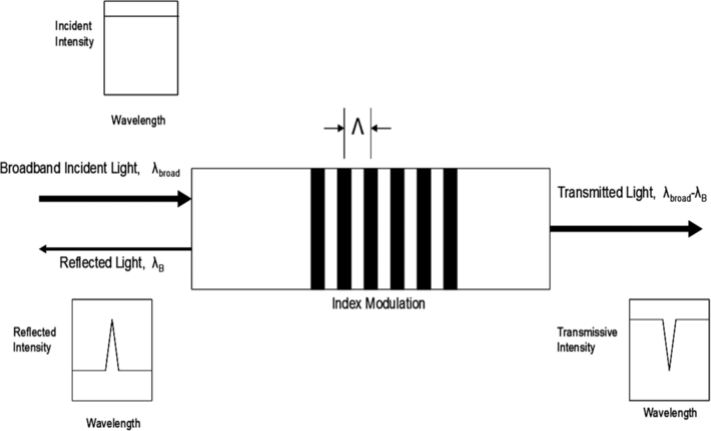
A fiber Bragg grating structure illuminated by broadband light.

Here, λ_B_ is the center wavelength of the reflected light, *n*_*eff*_ is the effective refractive index of the fiber core, and Λ is the grating spacing. Light that does not meet the Bragg condition transmits through the structure. The structure and illumination process are represented in Figure 2. In this work, λ_B_ ranged from 1540nm to 1560nm, *n*_*eff*_ was approximately 1.46, and Λ was approximately 530nm. Index modulations are in the 10^-4^ range and grating lengths range from 3mm to 10mm.

### Strain Sensitivity of FBG Sensor

Axial strain changes the length of the FBG of Δl and as a result produces a change in the grating period, Λ. In addition, axial strain induces a change in *n*_*eff*_, the effective index of the grating. It can be shown [18] that the change in the center wavelength due to axial strain is given by

(2) $$\Delta \lambda_{B}=2\left(\Lambda \frac{\partial n_{\mathrm{eff}}}{\partial l}+n_{\mathrm{eff}}\frac{\partial \Lambda }{\partial l}\right)\Delta l$$

which can be further simplified as

(3) $$\Delta \lambda_{B}=\lambda_{B}\left(1-p_{e}\right)\varepsilon_{z}$$

In Equation 3, *p*_*e*_ is the effective strain-optic coefficient and ε_z_ is the axial strain. The strain-optic coefficient, *p*_*e*_, is determined by

(4) $$p_{e}=\frac{n_{\mathrm{eff}}^{2}}{2}\left[p_{12}-v\left(p_{11}+p_{12}\right)\right]$$

where *p*_*12*_ and *p*_*11*_ are components of the strain-optic tensor and *v* is Poisson’s ratio.

The FBG sensor is extremely sensitive to axial strain. Small changes in the length of the fiber Bragg grating cause measurable changes in the reflected light spectrum. However, since a sensor that is implanted within a tendon will undergo a significant amount of compression loading due to the structure of the tendon and the plane of force exertion, this compressive load must be converted to axial loading on the fiber. To accomplish this, we integrated a bowed housing or a flexure mount onto the FBG. In the next section we describe the methods for designing, fabricating and testing this modified FBG sensor.

## Methods

### Sensor design

**Figure 3:**
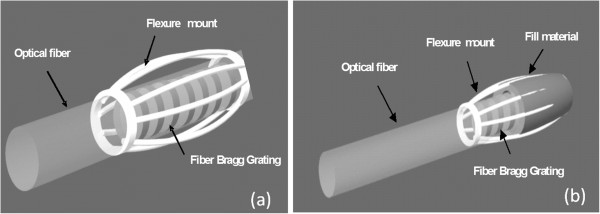
Flexure mount concept.

As discussed previously, the fiber Bragg grating is extremely sensitive to axial strain but must be modified to respond to the compressive loads within the tendon. This was accomplished with a bowed housing or a flexure mount as envisioned in Figure 3. The housing was hollow in the center to allow the fiber to pass through it. The lower limit for the housing inner diameter was defined by the cladding diameter of a single mode optical fiber, typically 125μm. The ends of the housing were fixed to the optical fiber, and the fiber Bragg grating was contained in the center. The struts were bowed so that radial forces would cause elongation of the struts and in turn, elongation of the fiber Bragg grating. Except for fabrication constraints, there is considerable design freedom because the curvature of the struts, the thickness and width of the strut material, and the elastic properties of the housing material all influence the relationship between radial forces and fiber elongation. Limiting the invasiveness of the sensor requires minimizing the outer diameter of the sensor while still allowing for axial loads to produce measurable shifts in the fiber Bragg grating center wavelength.

Notably, by monitoring the reflection from the grating, the sensor terminates at the end of the housing. This makes insertion easier than with other fiber optic methods that measure light transmission. Transmission measurements require that the fiber be inserted completely through the tendon and that each end of the fiber be polished to optical quality.

Moreover, it is possible to adjust the compliance of the sensor by including a fill material within the housing. This is illustrated in Figure 3b. The fill material increases the stiffness of the sensor and thus helps prevent permanent deformation or buckling of the struts. A rigid fill material like hard epoxy would be suitable for higher loads, while a flexible material like silicone would be useful for lighter forces.

### Sensor fabrication

The sensors shown in Figures 3a and b primarily consist of a bare fiber Bragg grating integrated with a custom made flexure mount. A variety of fiber Bragg gratings were obtained from a commercial vendor, Corvis Corporation, with center wavelengths varying from 1545nm to 1560nm. The length of the grating regions were all 5 mm. The largest challenge was fabricating the small flexure mounts. We investigated a number of different fabrication methods, however, the following two methods showed the most promise for producing reliable parts with consistent sizes and shapes: (1) hydroforming combined with laser micromachining, (2) swaging combined with laser welding and micromachining.

**Figure 4:**
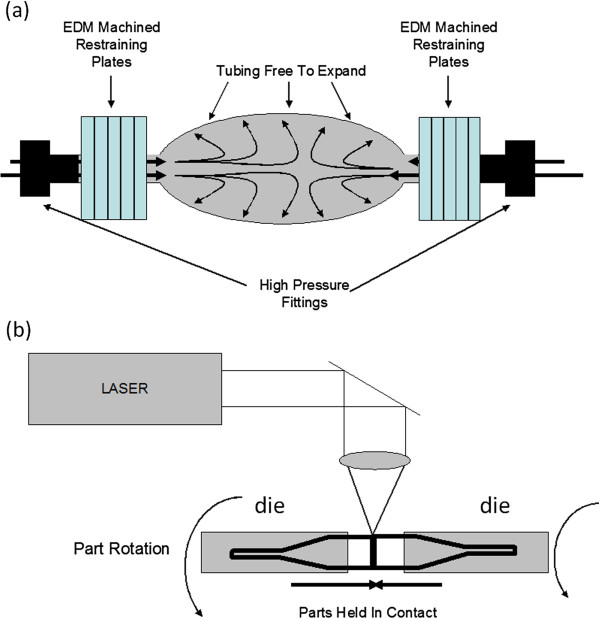
Swage and laser welding fabrication of flexure mounts.

As the name implies, hydroforming is a process in which a hollow structure, such as a tube, is inserted into a die and then pressurized by a fluid beyond its yield point to produce a shape that is determined by the die profile (see Figure 4a). After hydroforming a laser was used to micro-machine the struts.

Swaging is a forming technique in which the diameter of a rod, bar, or tube is reduced over a portion of its length in a tapered fashion [19]. Specifically, a work piece is rotated and fed into tapered dies that open and shut rapidly at rates that can exceed 1000 Hz. As the tubing is fed through, the die is inserted into a spindle and outer rollers on the spindle force the two halves together. These two halves are then laser welded together to form the part. As in hydroforming, once the surface profile is formed, the struts are fabricated by laser machining. The process and fixture are illustrated in Figure 4b.

**Figure 5:**
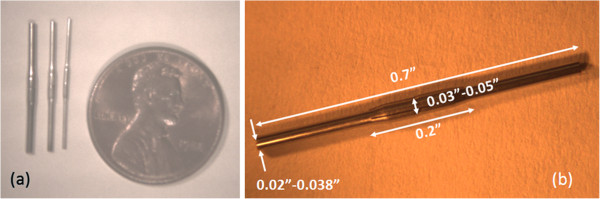
Typical flexure mount samples fabricated by swaging and laser welding.

While both methods were successful, the swaging process was the most suitable and produced the most consistent parts. Figure 5 shows several typical flexure mount parts that were fabricated via swaging, welding and laser machining. Parts with three different diameters of 0.02", 0.03" and 0.038" are illustrated from right to left in Figure 5a. Each part started with two 0.35" long sections of stainless steel tubing with wall thicknesses of 0.004". After swaging and laser welding, the parts were 0.7" long with bulged sections that were approximately 0.2" inches in length. The three different parts shown in Figure 5a had bulged sections whose centers were 0.05", 0.04" and 0.03" in diameter respectively. To complete sensor fabrication, we threaded the FBG fibers through the flexure mount devices and fixed them on both ends using standard epoxy. To vary the sensor’s overall stiffness, we filled the interior region of some sensors with a potting material, either silicon rubber or epoxy.

### Instrumentation

**Figure 6:**
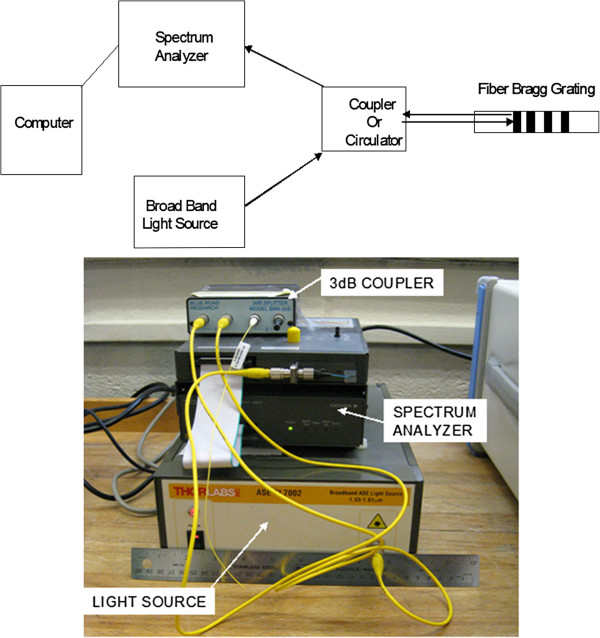
System instrumentation consisting of board band light source, optical beam delivery spectrum analyzer or optical channel monitor.

The overall sensing system, illustrated in Figure 6, comprised a broadband light source (ThorLabs ASE-FL002), beam delivery (Blue Road Research BRR-35S), and an optical channel monitor (Yokogawa FB200) that could measure the center wavelength of the light that was reflected from the grating. The whole system was controlled using a laptop computer running a Labview interface that communicated with the channel monitor via RS-232C. The FB200 was capable of reporting center wavelengths at rates up to 100 Hz. The Labview interface provided a real time display of the fiber Bragg grating center wavelengths and simultaneously wrote the wavelength values to a text file for further data processing. It should be noted that there are other optical configurations for interrogating an FBG sensor that may be more sensitive or cost-effective. For example, in [17] the authors employed a narrow band laser and single optical detector. In the future we intend to conduct a more comprehensive study on the advantages of different optical interrogation systems.

## Results

### Fiber bragg grating subjected to axial displacement

**Figure 7:**
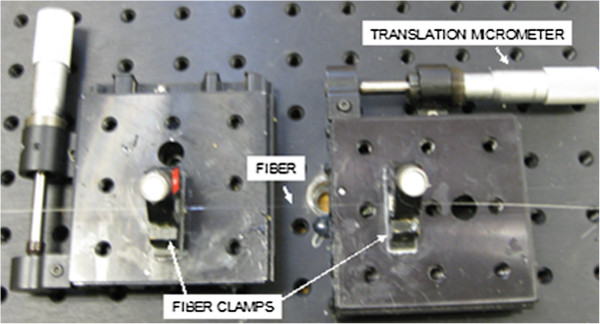
Fixture used to conduct axial displacement tests.

In order to verify that the fiber Bragg gratings and the optical instrumentation were functioning properly, we bonded a bare fiber Bragg grating (i.e. no flexure mount device) to fiber chucks, and mounted the fiber chucks to micrometer driven translation stages. We recorded the wavelength and original length between the bonded points. We then advanced the translation stage by 50 μm, held it in position, and repeated this process several times while the optical instrumentation and data acquisition were in operation. The fixture is shown in Figure 7.

**Figure 8:**
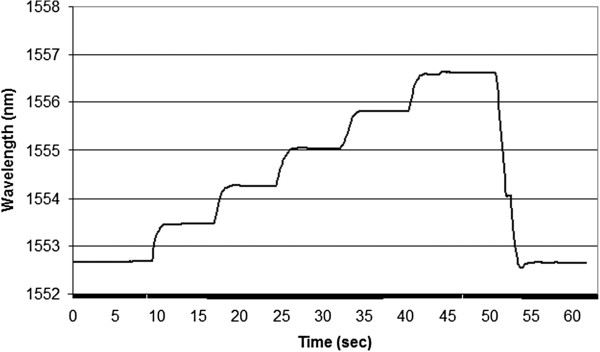
Wave length shift versus axial strain for 5 mm fiber Bragg grating.

Typical results from this experiment are shown in Figure 8. Here, each incremental step of 50 μm resulted in a calculated strain of 704 μstrain. By evaluating the change in wavelength per step, we determined an average value of 878 μstrain/nm for this set of data. These results lie within 7% of a theoretical value that is based strictly on the material properties.

### Hydrostatic testing

In order to better understand the behavior of the sensor, it is useful to test its performance under controlled conditions. We decided that a useful environment for the initial characterization of sensor performance would be uniform hydrostatic pressure.

**Figure 9:**
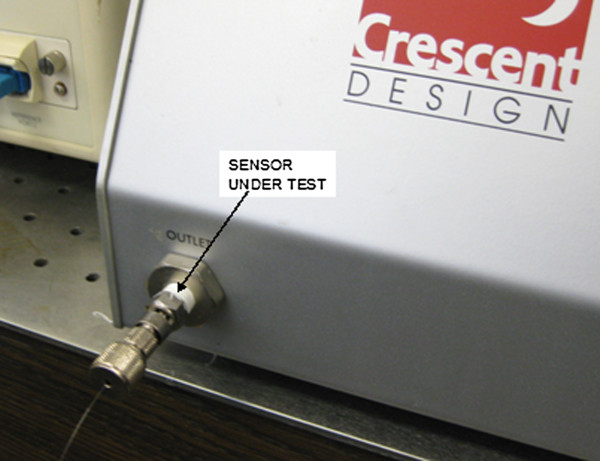
Sensor attached to HBLT-1000.

For this work, we selected a commercial hydraulic burst/leak tester (Model HBLT 1000) manufactured by Crescent Design, Inc. This device can produce programmable pressures from 0 to 1000 psi in 1.0 psi increments with 0.3 psi accuracy. Programmable profiles such as linear ramp, staircase, and fatigue are possible. This device is frequently used in the medical device and packaging industries for leak testing. A photograph of a mounted sensor is shown in Figure 9.

**Figure 10:**
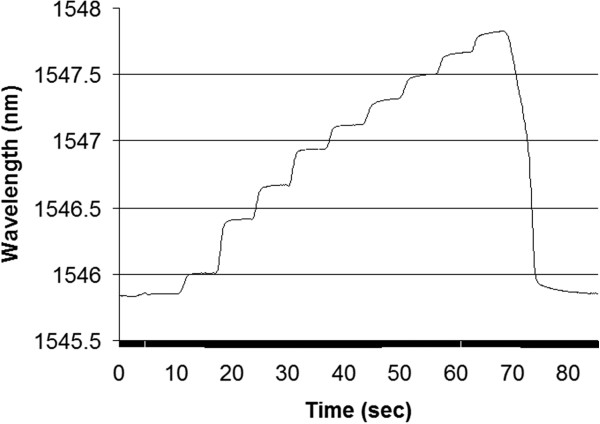
Stair case hydrostatic pressure testing, P= 10 psi to 100 psi in 10 psi steps.

Staircase testing was performed on the sensor. Here, the initial pressure was 10 psi and the staircase increment was 10 psi up to 100 psi. The pressure was maintained for 5 seconds between steps, and wavelength data was collected at 10 Hz. The trend line for the test is shown in Figure 10. The trend between steps was fairly consistent and the average wavelength shift per psi was in the range of 0.022 nm/psi. This indicates that experimentally, since the spectrum analyzer resolution is specified as 0.001nm, the pressure resolution of this sensor was less than 0.5 psi. The burst tester was unable to provide pressure increments of less than 1 psi.

**Figure 11:**
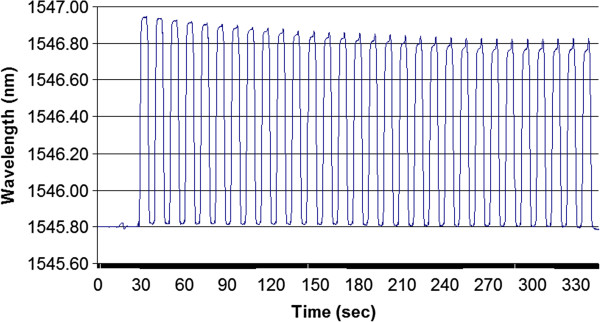
0 to 50 psi fatigue testing, 30 cycles were conducted.

Using this arrangement, we also performed fatigue testing on the sensor. Here, the sensor was taken through 30 cycles between 0 psi and 50 psi. Again the wavelength data was acquired at 10 Hz and is provided in Figure 11. As the data illustrates, there was some initial downward drift at the upper edge before the sensor stabilized, however this drift was small compared to the signal being measured. The calibration factor at the initial cycle was 0.022 nm/psi and at the 30^th^ cycle had only dropped to 0.020 nm/psi. Both numbers were very close to the staircase data. Thus, given the 3 psi specified accuracy of the burst tester, the performance was consistent.

### *In Vitro* testing

*In vitro* tests were completed to evaluate the sensor in representative tendon material.

### Characterization using excised deer tendon

**Figure 12:**
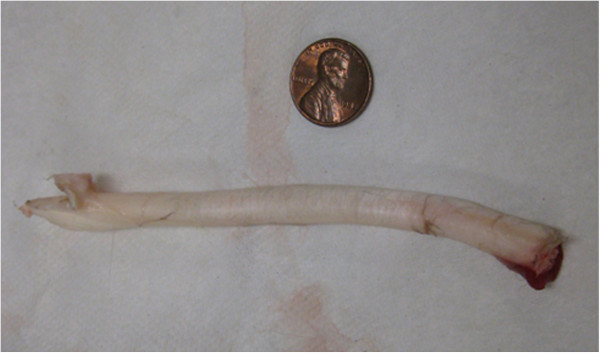
Hind leg deer tendon, removed for testing.

Tendons from the hind leg of whitetail deer were utilized for bench-top testing since they were readily available and their properties are close to human tendons such as the Achilles and Patella tendons. Since the tendon material consists of long strands, separate calibration data is needed to interpret the tendon forces. Figure 12 shows a single deer tendon that has been removed for testing.

**Figure 13:**
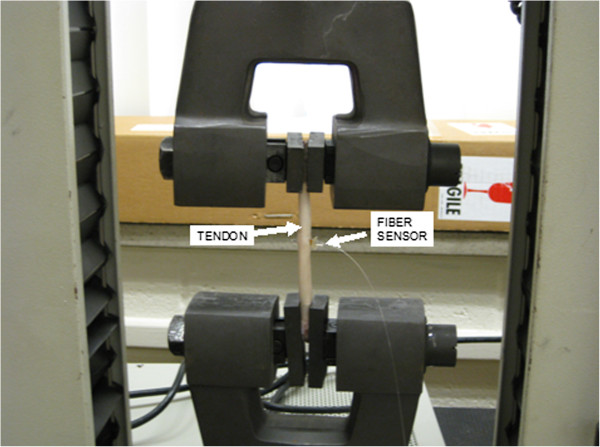
Tendon and fiber sensor in Instron tensile tester.

An Instron Universal Testing Instrument, Model 1011(tensile tester), was used to load the deer tendon during sensor evaluation. The tensile tester consisted of two clamps, a load cell, and a translation stage for applying tension and compression to the material being tested. A small incision was made in the tendon so that the fiber sensors could be inserted with a forced fit. Figure 13 shows a tendon mounted in the Instron for testing.

**Figure 14:**
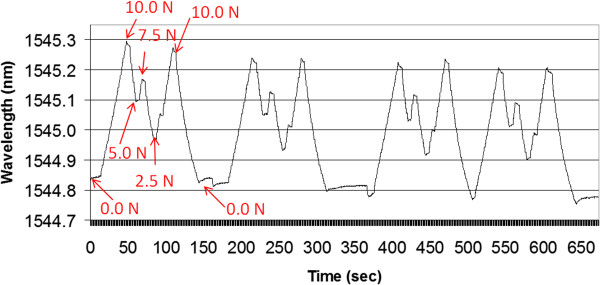
**Four consecutive Instron trials of the fiber sensor inserted within a deer tendon.** The force was cycled using the basic pattern 0, 10, 5, 7.5, 2.5, 5, 10, 0 N.

After several trials were made to determine the practical loading range of the tendon, we inserted the sensor and made a calibration run over the loading range. After testing, we made a second calibration run over the loading range. Figure 14 shows the results of 4 consecutive trials for a 4 strut, 6 mm long, 2.0 mm diameter, silicone filled sensor. The load cycle was 0 N, 10 N, 5 N, 7.5 N, 2.5 N, 5 N, 10 N, and 0 N.

**Figure 15:**
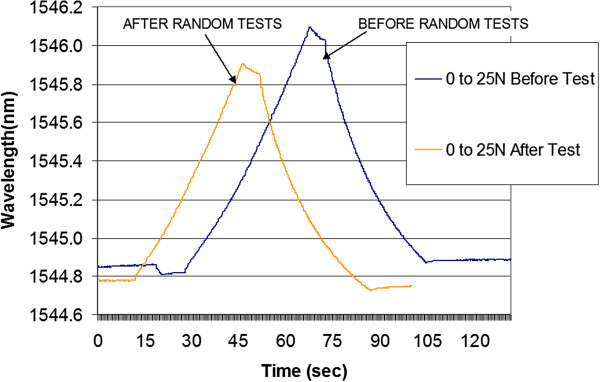
Results showing sensor output before and after calibration tests, 0 to 25 N.

From our data, we can make several interesting observations. First, the 5 N data point coming down from 10 N was higher than the 5 N data point coming up from 2.5 N. This held true for all 4 trials and relates to the tendon material relaxing in one case and increasing its tension in the other case. Second, there was a small reduction in the amplitude of the data points for each trial. This is likely associated with tissue damage in the clamps, fatigue in the tissue opening around the sensor, and hysteresis in the tendon material. This effect was also observed in the before and after calibration tests (Figure 15). Note that both the peak and the 0 N points have dropped after testing. In future testing we believe some of these effects can be reduced by using a modified tissue clamp. For example, other authors have had success mounting tendons using freeze clamps as described in [20].

### Characterization within a dynamic gait simulator

**Figure 16:**
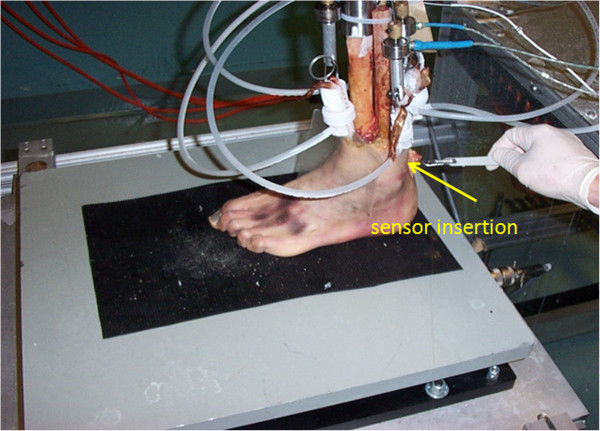
The Dynamic Gate Simulator, CELOS, Penn State University.

**Figure 17:**
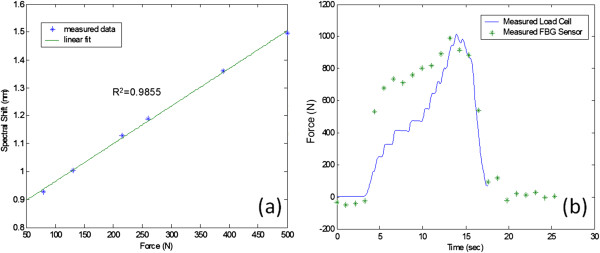
The Dynamic Gate Simulator results (a) 6 point calibration data and (b) results from 18 second walk cycle with load cell and optical sensor.

Additional tests were conducted at specialized facilities belonging to the Pennsylvania State University CELOS laboratory by invitation of Drs. Neil Sharkey and Stephen Piazza. The CELOS laboratory is equipped with a unique facility, the Dynamic Gait Simulator (DGS) [21], that produces in human cadaver specimens the time-scaled movements and ground reaction forces that occur during walking (Figure 16). Here, the sensors were used under realistic loading conditions and environments. In one test, a sensor was inserted along the medial-lateral axis of the Achilles tendon. Six data points were recorded for applied forces of 80 N, 130 N, 215 N, 260 N, 390 N, and 500 N (Figure 16). The foot was then taken through a time scaled walking cycle of 18 seconds, and synchronized data was recorded using both our optical sensor and the load cell on the DGS. Then, our wavelength data was converted to force data using the 6 point calibration curve, and was compared to the load cell data. As shown in Figure 17, there was reasonable agreement between the two profiles. We believe that some of the differences between the two profiles was due to tissue damage during sensor insertion. In the future we intent to employ a custom made cannula based insertion mechanism to reduce tissue damage. Additionally, in this particular test, the sensors exhibited saturation above 800 N. These sensors were filled with a hard epoxy in the hopes of increasing the dynamic range and thus, unfortunately lacked sensitivity. Regardless, our results indicate that these sensors function properly and can be extremely useful in biomechanics research settings. The use of human tissue during these experiments was reviewed and approved by Penn State's Institutional Review Board (IRB).

## Conclusions

In this paper we describe a fiber optic sensor that has been developed for the measurement of tendon forces. The sensor incorporates fiber Bragg gratings and micro-fabricated stainless steel housings. Stainless steel housings convert radial forces that are applied to the housing into axial forces that can be sensed by the fiber Bragg grating. This sensor offers a number of advantages over competing technologies including small size, high sensitivity, fast response time, large dynamic range and insensitivity to EMI.

Metal housings were fabricated using several methods including laser micromachining, swaging, and hydroforming. Swaging and hydroforming offered the best possibility of manufacturing large quantities of housing that have uniform dimensions and thus require less sensor-specific calibration.

A testing process was developed that allows for the study of sensors under controlled hydrostatic loading. Testing results indicate that these sensors can be produced with resolutions below 0.5 psi. These sensors were also successfully demonstrated in realistic *in vitro* testing conditions.

In the future, successful implementation of this sensor will require future study in several important areas. First, improved assembly methods will need to be developed. In particular these methods should be suitable for small housing dimensions and should result in more robust sensors. Along these lines, we are currently exploring the use of 3D printing as a means of fabricating consistent flexure mounts. Second, we are currently expanding our design to allow for distributed pressure sensing. In this design, multiple FBGs that are designed with different center wavelengths are distributed along the same optical fiber. This will allow sensing to take place at multiple locations with only a single optical feed. Third, we are investigating an introducer method that will make insertion of the sensor minimally invasive.

## Abbreviations

FBG: Fiber Bragg Grating; DGS: Dynamic Gate Simulator; EMI: ElectroMagnetic Interference.

## Competing interests

We have no conflict of interest to declare.

## Authors' contributions

All authors participated in conceiving the sensor and iterating on the design details. GB was the primary contributor in device fabrication, testing and data analysis. All authors have contributed towards drafting the manuscript and have, as such, read and approved the final manuscript.
